# Effects of the mycorrhizal fungus *Ceratobasidium* sp. AR2 on growth and flavonoid accumulation in *Anoectochilus roxburghii*

**DOI:** 10.7717/peerj.8346

**Published:** 2020-01-16

**Authors:** Ying Zhang, Yuanyuan Li, Shunxing Guo

**Affiliations:** Institute of Medicinal Plant Development, Chinese Academy of Medical Sciences & Peking Union Medical College, Beijing, China

**Keywords:** *Anoectochilus roxburghii*, *Ceratobasidium* sp., Growth indices, Flavonoid, Transcriptome, Ultra-high-performance liquid chromatography tandem mass spectrometry

## Abstract

**Background:**

*Anoectochilus roxburghii* is a traditional Chinese medicine with potent medicinal activity owing to the presence of secondary metabolites, particularly flavonoids. *A. roxburghii* also maintains a symbiotic relationship with mycorrhizal fungi. Moreover, mycorrhizal fungi can induce metabolite synthesis in host plants. However, little is known about the role of mycorrhizal fungi in promoting the accumulation of flavonoid metabolites in *A. roxburghii*.

**Methods:**

*A. roxburghii* and the isolated fungus *Ceratobasidium* sp. AR2 were cocultured. The portion of *A. roxburghii* above the medium treated with or without AR2 was studied by transcriptome and target metabolome analyses.

**Results:**

AR2 promoted the growth and development of *A. roxburghii*. The contents of total flavonoid, rutin, isorhamnetin, and cyanidin-3-glucoside chloride were increased compared with those in uninoculated cultures. Transcriptome analysis suggested that 109 unigenes encoding key enzymes were potentially associated with changes in flavonoids. Quantitative real-time polymerase chain reaction of fourteen flavonoid-related unigenes showed that most flavonoid biosynthetic genes were significantly differentially expressed between inoculated and uninoculated plantlets.

**Conclusion:**

The isolate AR2 could significantly promote the growth and development of *A. roxburghii* and the accumulation of flavonoids. Overall, our findings highlighted the molecular basis of the effects of mycorrhizal fungi on flavonoid biosynthesis in *A. roxburghii* and provided novel insights into methods to improve the yield and quality of *A. roxburghii*.

## Introduction

*Anoectochilus roxburghii* (Wall.) Lindl. (Orchidaceae), also known as the “king medicine” and “golden herb,” is a rare perennial herb that is mainly distributed in China, Indian, Nepal, Japan and Vietnam (Xu et al., 2016). In China, this herb is produced in Fujian, Zhejiang, Guizhou, and Taiwan provinces (Ye, Shao & Zhang, 2017). *A. roxburghii* is used not only in health food products, but also in traditional Chinese medicine. Pharmacological studies have indicated that the entire plant can be used as medicine and has many medicinal properties (Hsiao et al., 2011; Tang et al., 2018; Zeng et al., 2016). Additionally, studies of the chemical components of this plant have shown that *A. roxburghii* contains many bioactive substances, including flavonoids, polysaccharides, kinsenosides, steroids, triterpenes, amino acids, and alkaloids (Ye, Shao & Zhang, 2017). Among these compounds, flavonoids have been also used as a quality control index for *A. roxburghii*. To date, 26 main flavonoids and flavonoid glycosides have been identified in *A. roxburghii*, including quercetin, kaempferol, isorhamnentin and their glycosides (Ye, Shao & Zhang, 2017).

Flavonoids, which belong to a large family of polyphenols, are important plant secondary metabolites that can be classified into flavanones, flavones, flavonols, flavanols, isoflavones, and anthocyanidins according to their structure (Iwashina, 2000). Flavonoids possess a wide variety of biological functions in plants, such as attracting insects for pollination (Cortell & Kennedy, 2006; Wang et al., 2013), protecting plants against ultraviolet light (Dong et al., 2019; Yamaguchi, Kato & Di Mascio, 2009), and resisting pathogenic infections (Camargo-Ramírez, Val-Torregrosa & San Segundo, 2018; Steinkellner et al., 2007). Flavonoids are mainly used as food additives to increase the health functions of the foods owing to their many pharmacological effects, including antibacterial (Xie et al., 2015), anti-inflammatory (Ur Rashid et al., 2019), memory-enhancing (Ishola, Jacinta & Adeyemi, 2019), anti-Alzheimer’s disease (Ribaudo et al., 2019), antidiabetic (Da Silva et al., 2014), and antioxidant effects (Bubols et al., 2013). However, the flavonoid contents in *A. roxburghii* are very low, and wild *A. roxburghii* resources face extinction due to overexploitation and slow growth rates. This limits the development and utilization of the medicinal value of *A. roxburghii*.

Orchid mycorrhizal symbiosis plays an important role in the life cycle of orchids (Rasmussen, 2002; Warcup, 1981). Mycorrhizal fungal invasion and colonization not only promote growth and development, but also induce changes in the the internal metabolism of host plants (Stockel et al., 2014). Fungal elicitors can activate key enzymes or the expression of specific genes related to secondary metabolic pathways. Li et al. (2017a, 2017b) explained that *Mycena* sp. MF23 induced the expression of genes related to synthetic pathways to promote polysaccharide and dendrobine accumulation in *Dendrobium nobile*. These inducible metabolites are typically the major active ingredients in host plants, such as polysaccharides from *D. officinale* (Chen et al., 2016), dendrobines from *D. nobile* (Li et al., 2017a), and total flavonoids from *A. formosanus* (Zhang et al., 2013). For *A. roxburghii*, flavonoids are considered the basis of its important pharmacodynamic substances. Many studies have focused on increasing flavonoid contents in *A. roxburghii* by changing the external environment such as light quality (Wang et al., 2018; Ye et al., 2017). However, few reports have evaluated the effects of mycorrhizal fungi on flavonoid contents (Zhang et al., 2013).

In this study, a mycorrhizal fungus, *Ceratobasidium* sp. AR2, was isolated and identified from wild *A. roxburghii*. To better understand the effects of the AR2 isolate on *A. roxburghii* growth and flavonoid accumulation, we conducted cocultures and next-generation RNA sequencing (RNA-seq) analysis; the expression levels of genes in the flavonoid biosynthetic pathway and the contents of flavonoids were verified by quantitative real-time reverse transcription polymerase chain reaction (qRT-PCR) and ultra-high-performance liquid chromatography tandem mass spectrometry (UHPLC-MS/MS), respectively. This study aims to provide important insights into the role of the mycorrhizal fungus AR2 in improving the yield and quality of *A. roxburghii*.

## Materials and Methods

### Isolation of mycorrhizal fungi

The roots of wild adult *A. roxburghii* were collected from tree hoods under the mountain streams in Dehua County, Fujian Province, southeast China in November 2017. Mycorrhizal fungi were isolated following the methods of Hoang et al. (2017) and Richardson, Currah & Hambleton (1993), with some modifications. Roots were rinsed with tap water to remove soil and impurities, and then treated with 1% sodium hypochlorite disinfectant for 1 min. Roots were disinfected for 30 s with double antibiotics (penicillin–streptomycin, both at a final concentration of 50 μg/mL) and then rinsed three times for 1 min each in sterile distilled water. The root surface was scraped using an anatomical needle, and mycelium pelotons were then released into the sterile distilled water. The mycelium pelotons were transferred to a small piece of double-resistant potato dextrose agar (PDA) medium with a pipette gun under a GL6545 stereomicroscope (Guilin Guiguang Instrument Co. Ltd., Guangxi, China), and the plates were incubated in a dark incubator at 22 °C. Samples were then incubated until hyphae were visible on the medium. Pure culture was obtained by transferring the hyphae onto PDA. The isolated strains were then taken to the Institute of Medical Plant Development, Chinese Academy of Medical Sciences/Peking Union Medical College (maintained at 4 °C and −80 °C).

### Coculture of plant and mycorrhizal fungi

Tissue culture plantlets of *A. roxburghii* (approximately 3 months old and 2–3 cm in height) were used for coculture. Plantlets were transplanted into tissue culture bottles (9 cm in diameter, 12.5 cm in height) containing 125 mL H1 oat medium agar (H1OMA; 200 mg Ca(NO_3_)_2_·4H_2_O; 100 mg KCl; 200 mg KH_2_PO_4_; 100 mg MgSO_4_·7H_2_O; 100 mg yeast extract; 2 g sucrose; 3 g finely ground oats; 5 g agar; and 1 L H_2_O) (Clements, Muir & Cribb, 1986). Twelve bottles were used for each group (control group with no fungus and treatment group with fungus). One mycelial plug (approximately 1 cm × 1 cm) from 7-day-old fungi grown on PDA medium was placed into the center of each culture bottle, and four plantlets were then planted. The culture bottles were then kept in a conventional greenhouse with a 12-h light/12-h dark photoperiod at 24 °C ± 1 °C and an illumination intensity of 1,500 Lx. For contrastive evaluation of leaf color between the control and treatment groups, the RGB color pattern in Photoshop software was used to analyze the R, G, and B values of leaf color from both groups (Afshari-Jouybari & Farahnaky, 2011).

After 4 months, the root number, fresh weight, node number and tiller number of each plantlet were recorded. Section I tissues (the upper plantlets of the medium from the control group) and Section II tissues (the upper plantlets of the medium from the treatment group) were frozen with liquid nitrogen and stored at –80 °C until RNA-seq, qRT-PCR, and target metabolism analyses. Each treatment contained three replicates with six plantlets per replicate.

### Histological study

One week after inoculation, fresh root segments were fixed in a solution of 2.5% glutaraldehyde and 1.6% paraformaldehyde in 50 mM phosphate buffer (pH 6.8) for 4 h at room temperature. After fixation, the samples were washed three times with phosphoric acid buffer for about 15 min each time. The samples were then dehydrated through a graded ethanol series (15% ethanol for 30 min; 30% ethanol for 30 min; 50% ethanol for 30 min; 70% ethanol for 1 h; 85% ethanol for 1 h; 95% ethanol for 1 h; absolute ethanol for 1 h). After dehydration, the samples were embedded in LRwhite gradient mixture (25% LRwhite for 24 h; 50% LRwhite for 24 h; 75% LRwhite for 24 h; 100% LRwhite for 24 h) and polymerized for 48 h at 60 °C. Next, 3-µm-thick sections were cut using glass knives with a Reichert-Jung 2040 Autocut rotary microtome. The sections were collected on slides and stained with 0.05% (w/v) toluidine blue O in benzoate buffer for general histology examinations. The sections were examined, and images were captured digitally using a digital camera attached to the microscope (Axio Imager A1; Carl Zeiss, Oberkochen, Germany).

### Molecular identification of fungus

After 7 days in PDA medium, an E.Z.N.A. Fungal DNA Mini Kit (Omegabiotek, Norcross, GA, USA) was used to extract genomic DNA from mycelia. The internal transcribed spacer (ITS) regions were amplified using primers ITS1 (5′-TCCGTAGGTGAACCTGCGG-3′) and ITS4 (5′-TCCTCCGCTTATTGATATGC-3′) (Zhou, Tang & Guo, 2018). PCR was performed in a 50 μL reaction volume containing 2 μL template DNA (200 ng/μL), 1 μL each primer (10 μM), 25 μL Taq PCR mix, and 21 μL sterile water. The reactions were performed in a Bio-Rad T100 Thermal Cycler (Bio-Rad, Hercules, CA, USA) using the following program: initial denaturation at 95 °C for 3 min; 35 cycles of denaturation at 94 °C for 1 min, annealing at 55 °C for 30 s, and extension at 72 °C for 1 min; and a final extension at 72 °C for 7 min. Amplification products were analyzed in 1% agarose gels (mixed with golden view) by electrophoresis and directly sequenced using a GENEWIZ sequencer (Suzhou, China). The sequence was submitted to the GenBank database (Accession Number: MN068847). For phylogenetic analysis, ITS sequences of the AR2 isolate and 18 *Ceratobasidium* sequences of known species from GenBank were analyzed, and the sequence of *Waitea circinata* was used as the outgroup taxon (Paduano et al., 2011). DNA sequences were aligned using Clustal W algorithm in MEGA 7.01, followed by manual adjustment. Distance trees were obtained using the neighbor-joining (NJ) method (Saitou & Nei, 1987) with the Tajima-Nei method. For assessing the relative robustness of branches, the bootstrap method was used with 1,000 replicates (Felsenstein, 1985).

### Total RNA extraction

Total RNA from each sample was extracted with an RNeasy Plant Mini Kit (Qiagen, Hilden, Germany) according to the manufacturer’s instructions, followed by treatment with an RNase-free DNase I digestion kit (Aidlab, Beijing, China) to remove genomic DNA contamination. RNA quality and concentration were verified by 1% agarose gel electrophoresis and NanoDrop 2000 spectrophotometry (Thermo Fisher Scientific, Waltham, MA, USA), respectively. RNA integrity was assessed on an Agilent 2100 Bioanalyzer (Agilent Technologies, Santa Clara, CA, USA). Library construction and sequencing were performed on an Illumina Hiseq Xten platform. Sequence data were deposited in the NCBI database (Accession Number: PRJNA562071).

### cDNA library construction, sequencing, assembly and annotation

mRNA was isolated from total RNA with oligo (dT)-attached magnetic beads and randomly fragmented into short fragments using fragmentation buffer. With cleaved RNA fragments as template, the first cDNA strand was synthesized by random hexamers, and the second cDNA strand was synthesized by adding buffer, dNTPs, RNaseH, and DNA polymerase I. Double-stranded cDNA was then purified using a QIAEX II Gel Extraction kit (Qiagen). The ends were trimmed, and a poly (A) tail was added at the sequencing joint. PCR amplification was then performed, and PCR products were purified using an AMPure XP Kit (Beckman Coulter, Brea, CA, USA). Library quality was assessed on an Agilent 2100 Bioanalyzer (Agilent Technologies, Santa Clara, CA, USA).

Deep transcriptome sequencing was performed using the Illumina Hiseq Xten. Raw data were filtered by removing the connector sequence and low-quality reads to yield high-quality clean data. Sequence alignment was conducted between the clean data for each sample and a transcript or unigene library was assembled. The obtained transcript and unigene reads were categorized as mapped reads and were used for subsequent analysis. Unigene function was annotated based on the following six databases: NCBI non-redundant protein sequences (Nr), NCBI non-redundant nucleotide sequences (Nt), Clusters of Orthologous Groups of proteins (COG), a manually annotated and reviewed protein sequence database (Swiss-Prot), Kyoto Encyclopedia of Genes and Genomes (KEGG), and Gene Ontology (GO).

### Confirmation of flavonoid biosynthesis-related genes

Unigenes related to flavonoid biosynthesis were validated with qRT-PCR. RNA from the two groups (I and II) was reverse-transcribed into cDNA with a PrimeScript RT reagent kit (TaKaRa Bio, Shiga, Japan). qRT-PCR was performed with SYBR Premix Ex Taq (TaKaRa Bio, Shiga, Japan) on a LightCycler 480 II Real-Time PCR System (Roche Diagnostics, Basel, Switzerland). Three biological replicates and three technical replicates were analyzed, and all primer names and corresponding sequences are shown in Table S1. The elongation factor 1 alpha (*EF-1α*) gene in *A. roxburghii* was used as a reference control. The PCR conditions were as follows: denaturation at 95 °C for 30 s, followed by 40 cycles of amplification (95 °C for 5 s, 60 °C for 30 s). Melting curves were prepared by measurement at 95 °C for 5 s and 60 °C for 1 min. For each sample, three technical replicates were used, with a minimum of three biological replicates. Gene expression was evaluated using the 2^−ΔΔCt^ method (Livak & Schmittgen, 2001).

### Extraction and analysis of total and main flavonoids

Flavonoids were extracted using an ultrasonic-assisted method. Each sample (approximately 20 mg) was soaked in 400 μL of 90% methanol for approximately 2 h according to the water-to-raw material ratio (20 mL/g) and then extracted three times for 20 min each time in an ice-water bath using the KH5200DE ultrasonic instrument (Kunshan Hechuang Ultrasonic Machinery Co., Jiangsu, China). Samples were then centrifuged at 10,000×*g* for 5 min, and each extract was collected and pooled.

The contents of total flavonoids were determined using colorimetry. Briefly, 150 μL of 5% NaNO_2_ was added to one mL extract in a 10 mL volumetric tube, and the mixture was incubated for 6 min at room temperature. Next, 150 μL of 10% AlCl_3_·6H_2_O was added to the mixture, and the mixture was incubated for an additional 6 min, followed by the addition of 2 mL of 4% NaOH. After 15 min of incubation at room temperature, the absorbance at 510 nm was measured using an EnSpire Multimode Plate Reader (PerkinElmer, Waltham, MA, USA). Total flavonoid contents were calculated using a standard rutin curve. The linear regression equation was *y* = 1.9322*x* + 0.0501 (correlation coefficient (*R*^2^) = 0.9997).

To determine changes in monomer flavonoids, several available flavonoids known to be present in *A. roxburghii* were tested. Rutin (153-18-4; 98% purity), quercetin (117-39-5; 98% purity), isorhamnetin (480-19-3; 98% purity), isorhamnetin-3-o-rutinoside (604-80-8; 98% purity), kaempferol (520-18-3; 98% purity), and cyanidin-3-glucoside chloride (7084-24-4; 98% purity) were purchased from National Institutes for Food and Drug Control (Beijing, China). Measurement of the concentrations of these flavonoids was performed using a 1290 Infinity II series UHPLC System connected to the 6460 Triple Quadrupole Mass Spectrometer (Agilent Technologies, Santa Clara, CA, USA). UHPLC separation was completed using an Agilent 1290 Infinity II series UHPLC System equipped with a Waters ACQUITY UPLC HSS T3 column (100 × 2.1 mm, 1.8 μm). The mobile phase A was 0.1% formic acid in water, and the mobile phase B was methanol. The elution gradient was as follows: 0–1 min, 80–80% A; 1–9.5 min, 80–5% A; 9.5–11.2 min, 5–5%; 11.2–11.5 min, 5–80%; 11.5–15 min, 80–80%. The flow rate was 0.3 mL/min, and the injection volume was 1 μL. The column and autosampler temperatures were 35 °C and 4 °C, respectively. An Agilent 6460 triple quadrupole mass spectrometer (Agilent Technologies, Santa Clara, CA, USA) equipped with an AJS electrospray ionization interface was used for assay development. Typical ion source parameters were as follows: capillary voltage = +4,000/−3,500 V, nozzle voltage = +500/−500 V, gas (N_2_) temperature = 250 °C, gas (N_2_) flow = five L/min, sheath gas (N_2_) temperature = 250 °C, sheath gas flow = 11 L/min, nebulizer = 45 psi. The six target flavonoids were used to obtain an external calibration curve, which was used for calculating the content of each flavonoid in each sample.

### Statistical analysis

SPSS 12.0 software (IBM, Chicago, IL, USA) was used for data analysis, and values are presented as the mean ± standard deviation. An independent Student’s *t*-test was used to compare the effects of mycorrhizal fungi on the response variables considered in this study. A value of *P* ≤ 0.05 was considered statistically significant.

## Results

### Fungal isolation and identification

**Figure 1:**
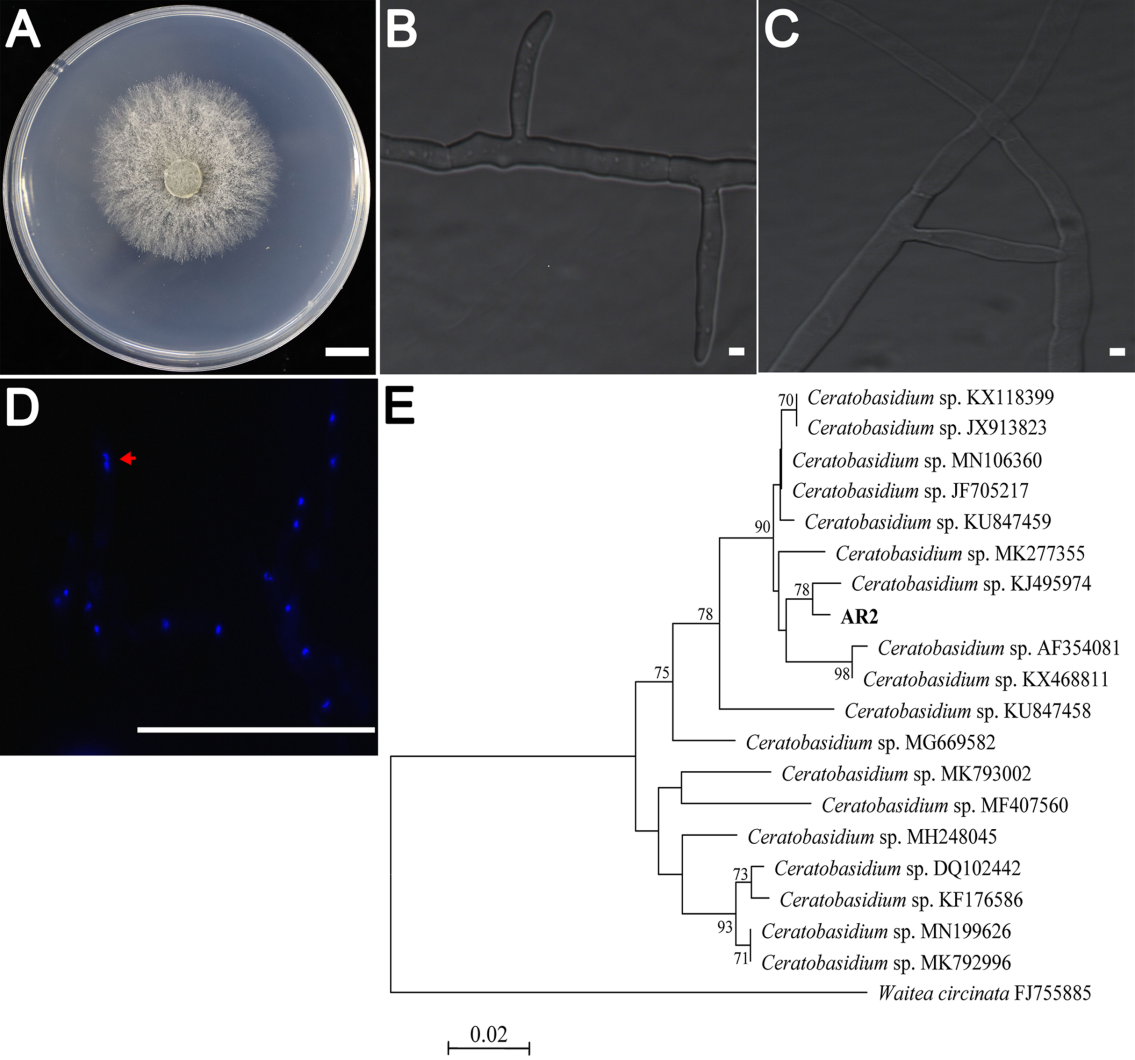
Morphological characters of the AR2 isolate. (A) Culture of isolate AR2 for 3 days on PDA, scale bar = 1 cm. (B) The morphological characteristics (straight-angle branching and hyphae constriction at branching points) of mycelia of AR2 under optical microscope, scale bar = 5 μm. (C) The morphological characteristics (hyphae fusion) of mycelia of AR2 under optical microscope, scale bar = 5 μm. (D) 4′,6-diamidino-2-phenylindole (DAPI)-stained hyphae of AR2 showing binucleate cells; the red arrow represents a binucleate in a mycelial cell; bar = 100 μm. Morphological observations of mycelium were performed using the fluorescence microscope, Zeiss AxioImagerA1 (Zeiss, Germany). (E) Neighbor-joining phylogenetic tree of ITS rDNA sequences from AR2 and 18 *Ceratobasidium* sequences of known species from GenBank. Bootstrap values (calculated from 1,000 resamplings) of above 70% are shown at the branch point. *Waitea circinata* is used as the outgroup.

AR2 cultures showed rapid growth (9–10 mm/day) on PDA medium. The colonies were yellowish-white in color, and the mycelium grew densely (Fig. 1A). Colony microstructure observations showed that the mycelia had right-angled branches and hyphae constriction at branching points (Fig. 1B). There was mycelial fusion between hyphae: short lateral branches were produced between two hyphae, and short lateral branches grew in opposite directions to complete mycelial fusion (Fig. 1C). *Ceratobasidium* and *Thanatephorus* are both members of genus *Ceratorhiza* (Dearnaley, Martos & Selosse, 2012). The main difference between these organisms is that the mycelial cells of the former are binucleate, whereas those of the latter are multinucleated. Our observation of the AR2 nuclear chamber showed that its hyphal cells were binucleate (Fig. 1D). A BLAST search of the ITS sequences based on the extracted rDNA showed 98% identity with the ITS sequences of *Ceratobasidium* sp. Moreover, the results of the NJ phylogenetic tree analysis showed that AR2 was closely related to *Ceratobasidium* sp. and aggregated into the same branch with *Ceratobasidium* sp. (KJ495974) isolated from *A. formosanus* (Jiang et al., 2015) (Fig. 1E). Thus, AR2 isolated from the roots of wild *A. roxburghii* was identified to be a member of the genus *Ceratobasidium* based on morphological features and phylogenetic tree analysis of the ITS sequences.

### Variations in biomass parameters in *A. roxburghii*

**Figure 2:**
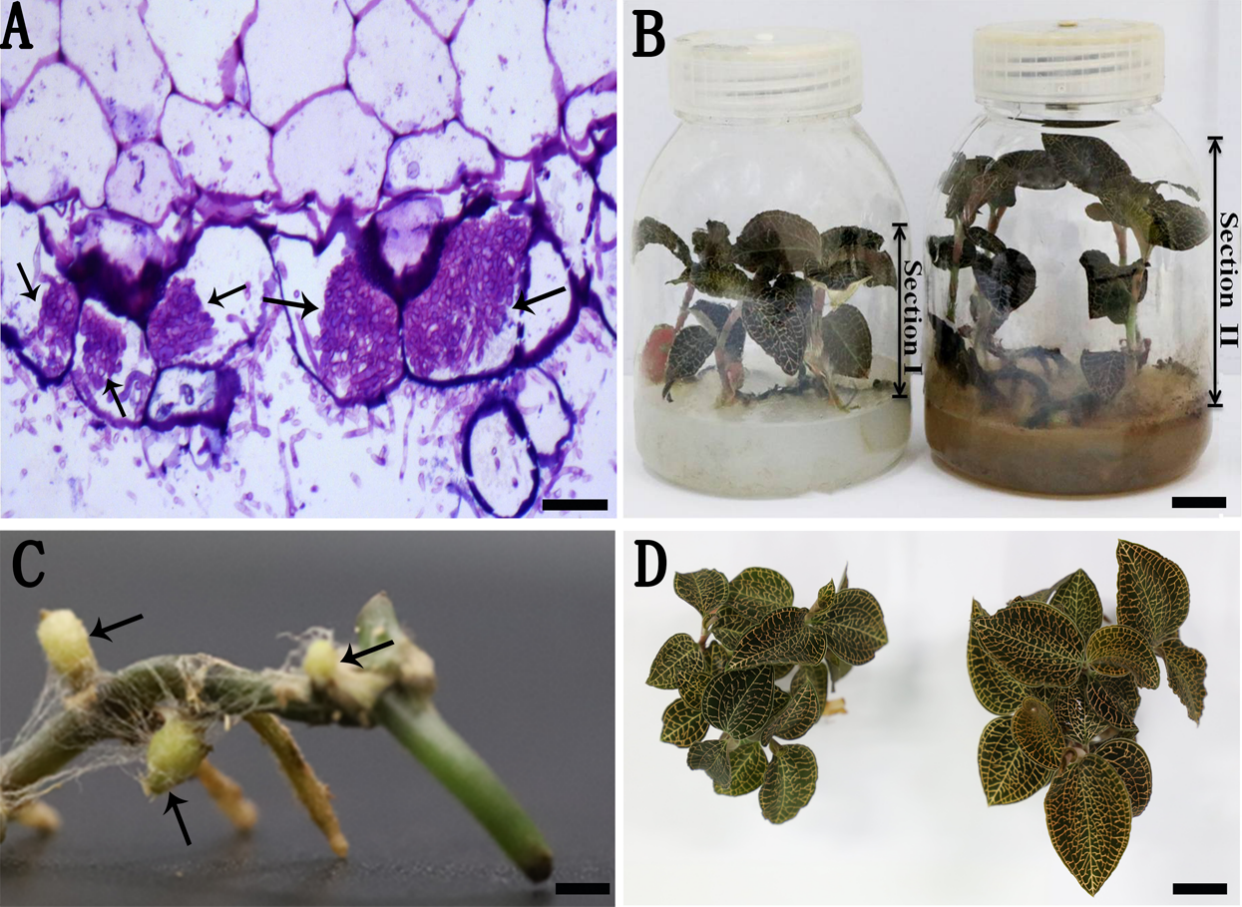
*A. roxburghii* inoculated with AR2 and uninoculation. (A) The semi-thin section of root of *A. roxburghii* after 1 week of symbiotic cultivation; arrows represent peloton, scale bar = 50 μm. (B) *A. roxburghii* grown in tissue culture bottle for 4 months inoculated with AR2 (Section II) and uninoculation (Section I). Section I represents the upper plantlets of the medium for the control; Section II represents the upper plantlets of the medium for the treatment; scale bar = 1 cm. (C) Local morphology from the plantlet of the treatment group; arrows represent new buds, scale bar = 1 cm. (D) Contrast of leave color between the control group (the left) and treatment group (the right) after 4 months of culture, scale bar = 1 cm.

After 1 week of coculture, the roots in the AR2 treatment group showed a typical structure (intracellular pelotons) of orchid mycorrhizae (Fig. 2A). After 4 months, the treatment group showed better growth than the control group (Fig. 2B), and new buds appeared in the treatment group (Fig. 2C). Additionally, the leaf color differed significantly between the two groups; the leaves of mycorrhizal *A. roxburghii* were redder than those of nonmycorrhizal plants (Fig. 2D; Table S2). The growth indices of *A. roxburghii* infected with AR2 are shown in Table 1. The fresh weight, dry weight, height, root number, and bud number were higher in the mycorrhizal plants than those in the control plants (Table 1).

**Table 1:** Effects of mycorrhizal fungi AR2 on growth of *A. roxburghii*.

	Fresh weight (g)	Dry weight (g)	Root number	Bud number	Height (cm)	Node number
ck	1.30 ± 0.21^b^	0.13 ± 0.03^b^	3.50 ± 0.67^b^	0.41 ± 0.51^b^	4.01 ± 0.66^b^	7.58 ± 0.67^a^
Treatment	1.90 ± 0.34^a^	0.31 ± 0.08^a^	4.67 ± 1.07^a^	3.33 ± 0.78^a^	5.45 ± 0.29^a^	8.08 ± 0.67^a^

**Note:**

Each value represents the mean ± standard deviation (SD) (*n* = 12). Different letters represent significant differences (*P ≤* 0.05) on the same row according to the independent-sample *t* test.

### Transcriptome sequencing, de novo assembly and unigene annotation

Orchidaceae mycorrhizal infection is often confined to underground roots and rhizomes (Singh & Varma, 2000). In order to avoid the interference of genes and compounds from mycorrhizal fungi, we chose uninfected portions of *A. roxburghii* above the culture medium for analysis. cDNA libraries were constructed using Section II of *A. roxburghii* inoculated with AR2 and Section I of normal *A. roxburghii* as a control (Fig. 2A). We sequenced and pooled equivalent quantities of RNA extracts isolated from the two sections, and all samples were sequenced in three biological replicates. After removing adaptors, ambiguous nucleotides, and low-quality sequences, 247.76 Mb clean reads (total data: 37.17 Gb) were obtained. Q20 and Q30 base percentages were greater than or equal to 95.68% and 89.28%, respectively. The GC contents of Section I and Section II were in the ranges of 47.62–47.74% and 48.76–49.00%, respectively (Table S3). The assembled sequences showed that the unigenes were in the range of 61,217–67,640 bp with an N50 length of 1,242–1,440 bp and 42.81–44.02% GC content, whereas transcripts were in the range of 92,355–105,866 bp with an N50 length of 1,140–1,330 bp and 42.79–44.07% GC content (Table S4). These data indicated that the constructed transcriptome library had high-quality sequencing data and could be used for subsequent analyses, providing abundant data resources for subsequent gene research.

**Figure 3:**
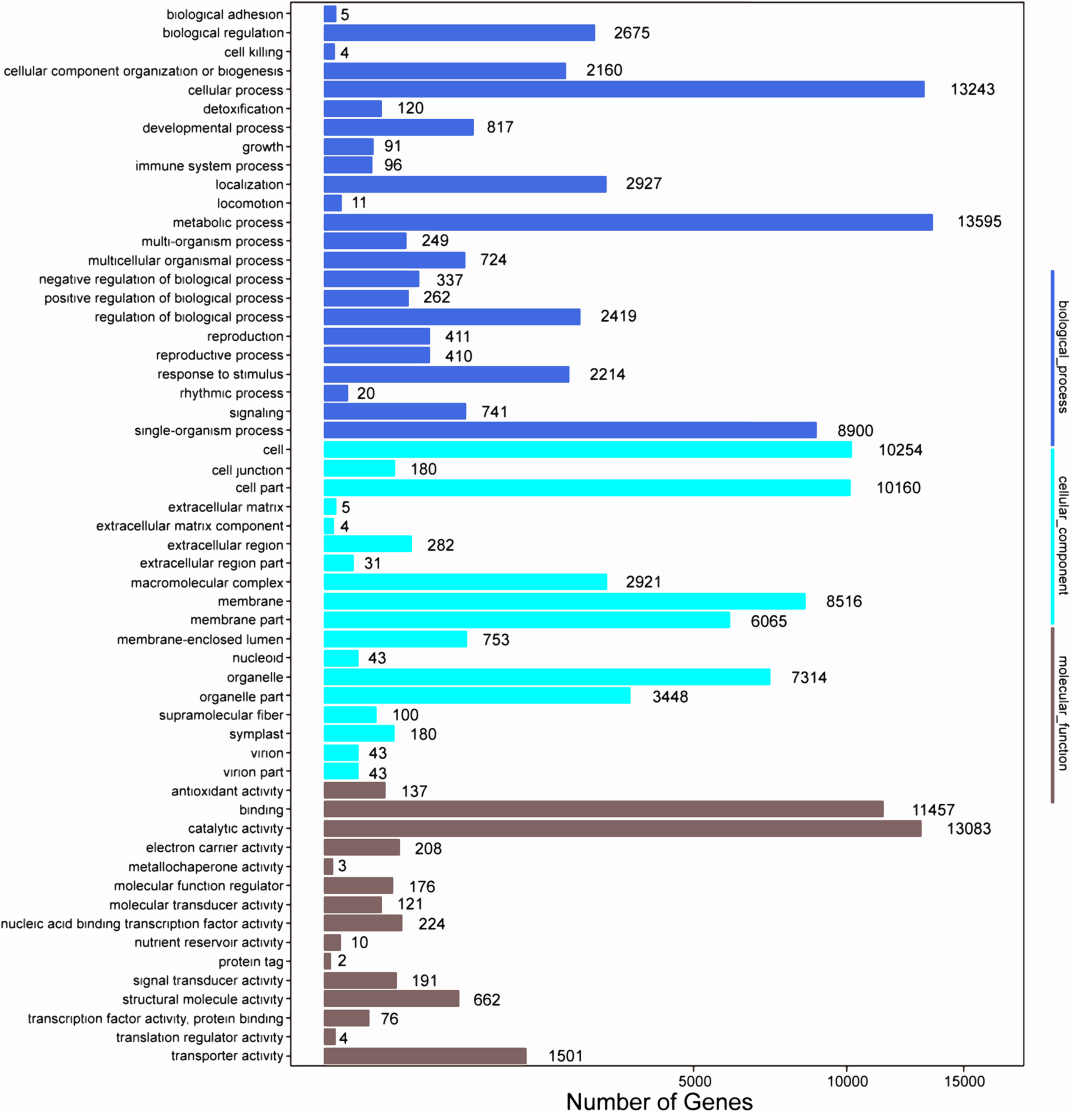
GO functional classification map of the unigenes.

Among the 133,321 identified unigenes, 63,136 (47.36%), 31,497 (23.62%), 46,082 (34.56%), 45,505 (34.13%), 25,349 (19.01%) and 26,447 (19.84%) were aligned to the Nr, Nt, SwissProt, KEGG, COG, and GO databases, respectively (Table S5). According to GO analysis, 130,618 non-redundant unigenes were classified into three major functional ontologies: biological process, cellular component, and molecular function (Fig. 3). For biological process, the dominant subcategories were cellular process (13,243) and metabolic process (13,595). In the category of cellular component, cell (10,254), cell part (10,160), and membrane (8,516) were the most highly represented. Among molecular function terms, binding (11,457) and catalytic activity (13,083) were the most highly represented. Moreover, within each of these three categories, a few genes were assigned the subcategories of “cell killing,” “extracellular matrix component,” and “protein tag.”

**Figure 4:**
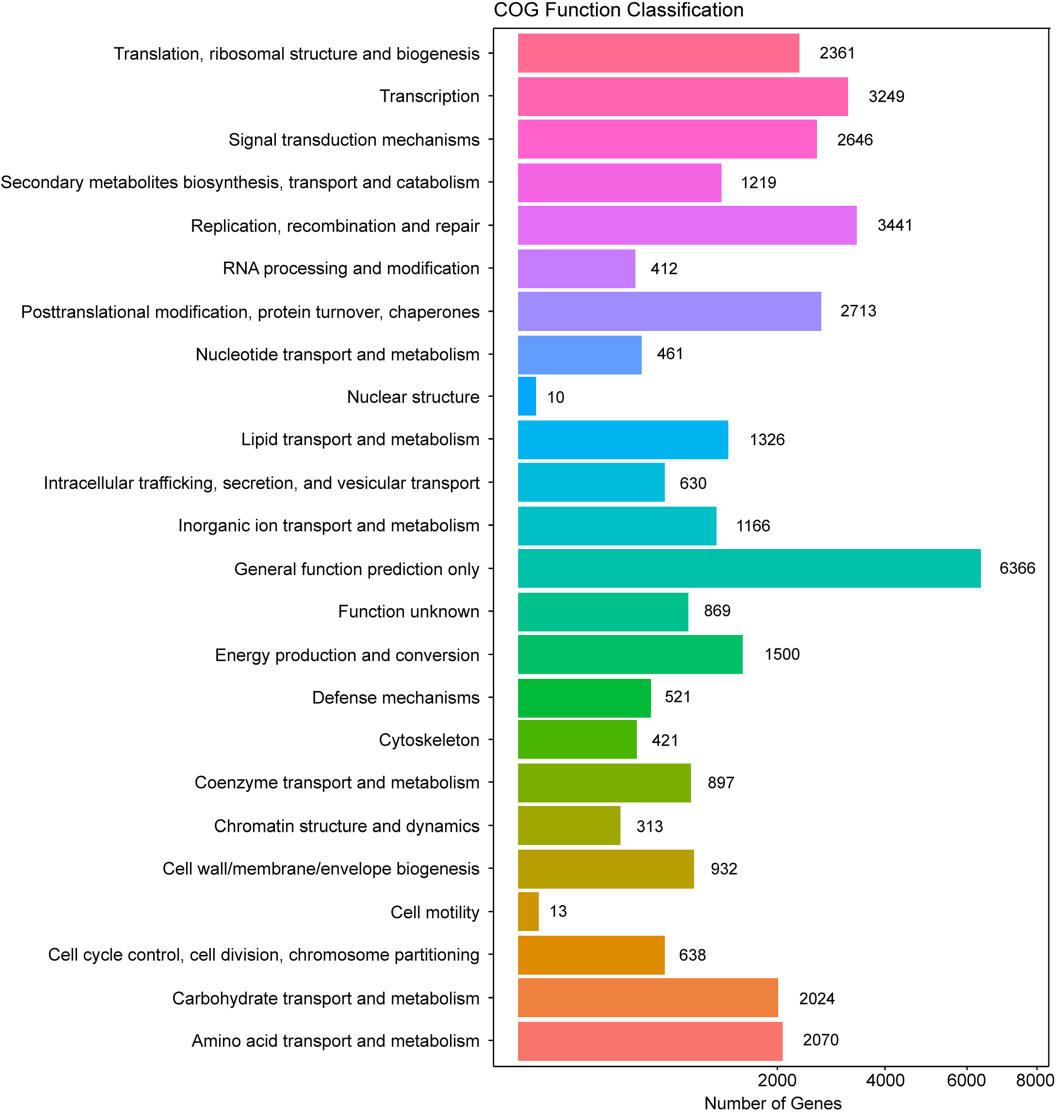
COG functional classification map of the unigenes.

In addition, all unigenes were searched against the COG database for functional prediction and classification. In total, 36,198 nonredundant unigenes were assigned to COG classifications and clustered into 24 functional categories (Fig. 4). Among these, the cluster of general function prediction only (6,366) was the largest group, followed by replication, recombination, and repair (3,441); transcription (3,249); post-translational modification, protein turnover, and chaperones (2,713); and signal transduction mechanisms (2,646). Only a few unigenes were assigned to the subcategories of cell motility (13) and nuclear structure (10). Furthermore, 869 genes were classified as having unknown functions. These GO and COG annotations showed that the expressed unigenes in *A. roxburghii* encoded many metabolism-related proteins.

**Figure 5:**
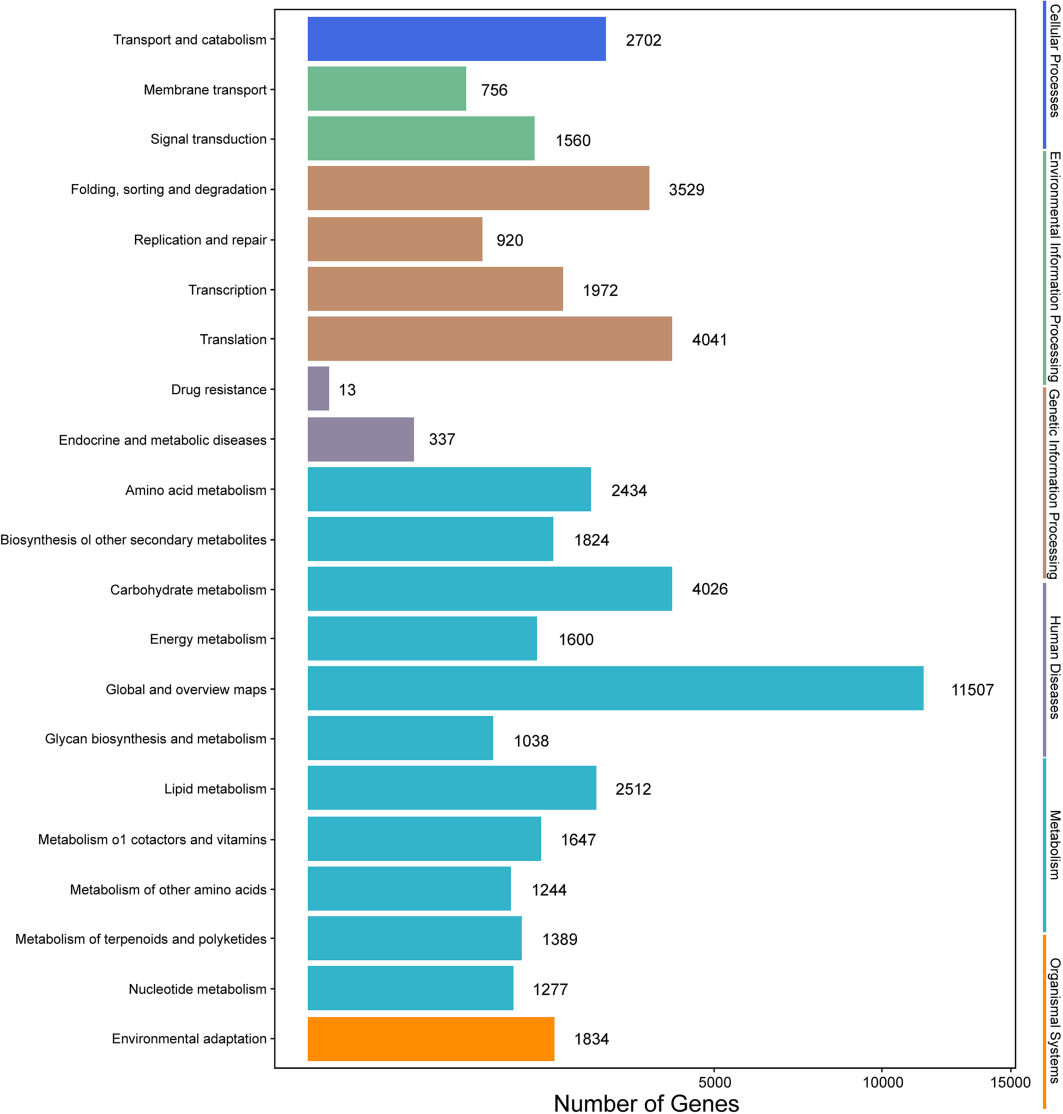
KEGG annotation of putative proteins.

Analysis of the metabolic pathways modulated by unigenes was performed using KEGG annotations. According to KEGG analysis, 68,046 unigenes were assigned to different pathways (Fig. 5). The results showed that metabolic pathways were the most enriched, followed by “genetic information processing.” The subcategory of human diseases was the least enriched. Flavonoid (ko00941), flavone and flavonol (ko00944), and anthocyanin metabolism (ko00942) were among the detected metabolic pathways, and 109 unigenes encoding proteins involved in flavonoid biosynthesis were identified (Table S6), indicating that the expression levels of these genes could be responsible for flavonoid accumulation.

### Analysis of differentially expressed genes

**Figure 6:**
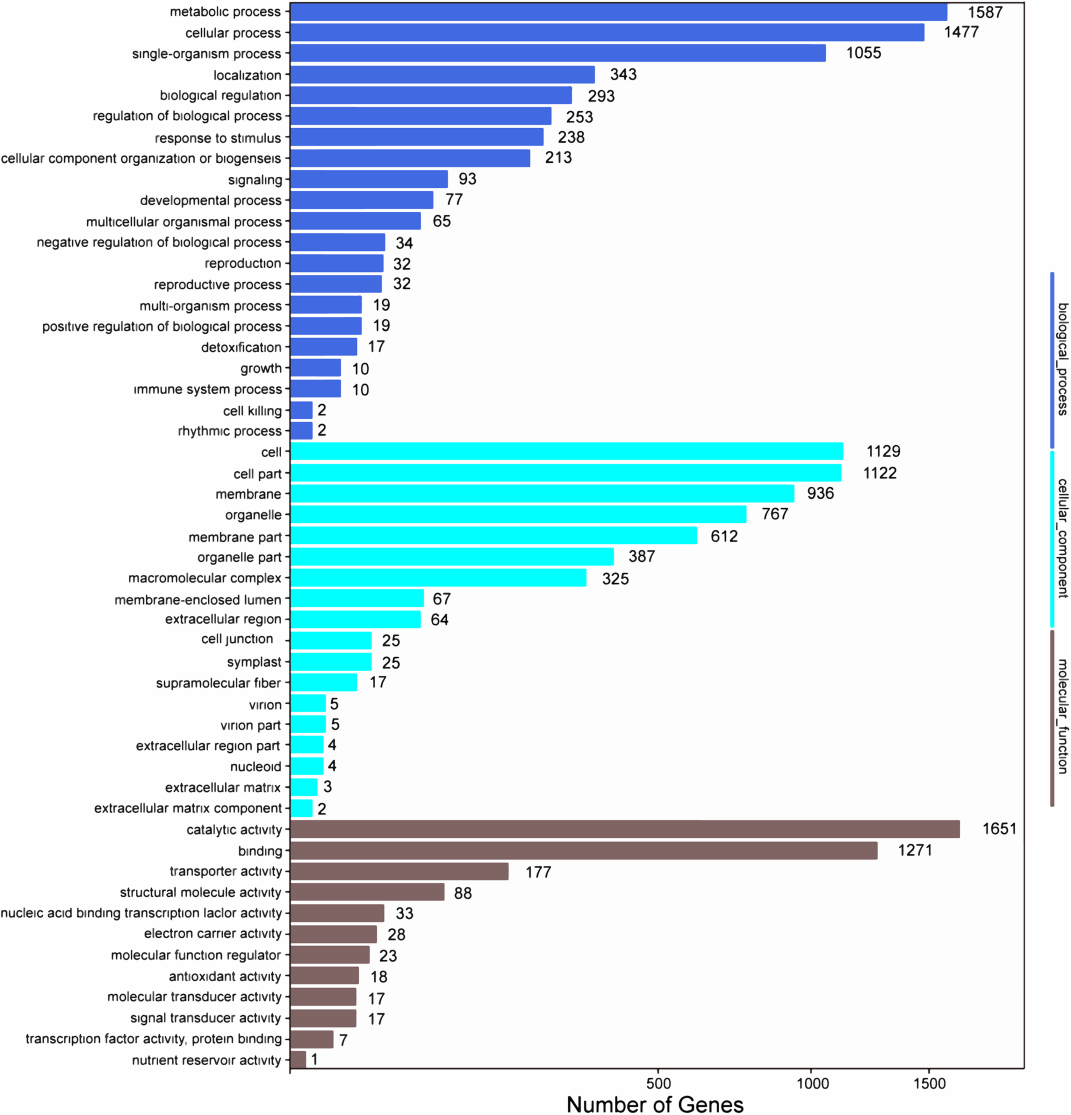
Go functional enrichment of the DEGs.

**Figure 7:**
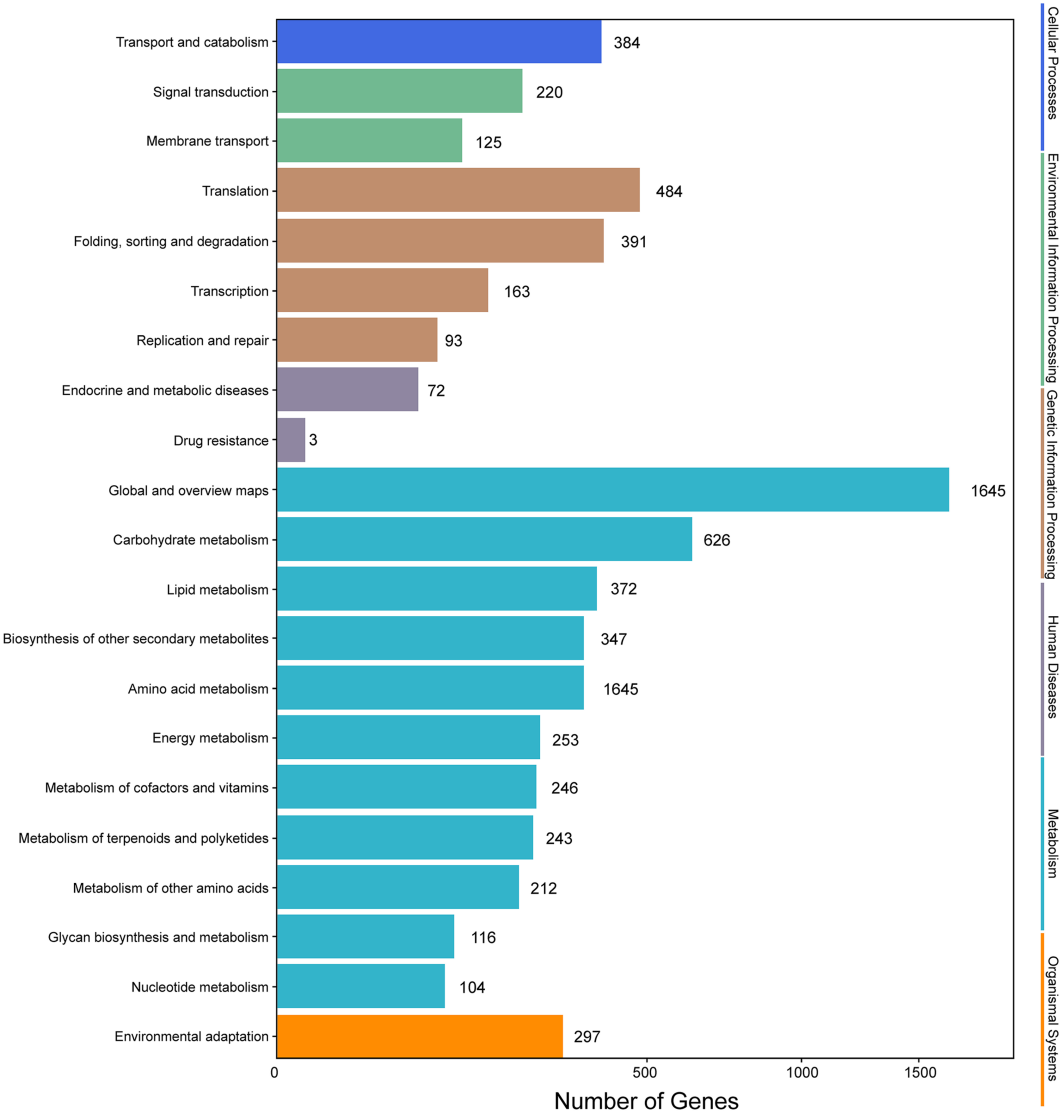
KEGG annotation of the DEGs.

Genes with |log_2_(fold change)| > 1 and *q* < 0.001 were defined as differentially expressed genes (DEGs). In this study, the six samples were divided into two groups, that is, groups I (I1, I2, I3) and II (II1, II2, II3). The different expression patterns of genes were then analyzed between groups I and II. In total, 9,645 DEGs were identified between groups I and II, with 6,223 up-regulated genes and 3,422 down-regulated genes. GO annotations showed that 14,669 genes were classified into 50 GO terms (Fig. 6), with metabolic process and catalytic activity as the dominant terms. KEGG pathway analysis of the DEGs from groups I and II revealed 136 enriched pathways. These pathways were involved in six processes, including cellular processes, environmental information processing, genetic information processing, human diseases, metabolism, and organismal systems (Fig. 7). In the top 20 pathways, the number of unigenes involved in metabolic pathways was higher (Table S7). Moreover, 23.90% and 14.95% of the top 20 KEGG pathways were involved in metabolic pathways and biosynthesis of secondary metabolites, respectively. These results suggested that AR2 had important effects on the metabolism of secondary metabolites in *A. roxburghii*.

### Relative expression of flavonoid synthesis-related genes

**Figure 8:**
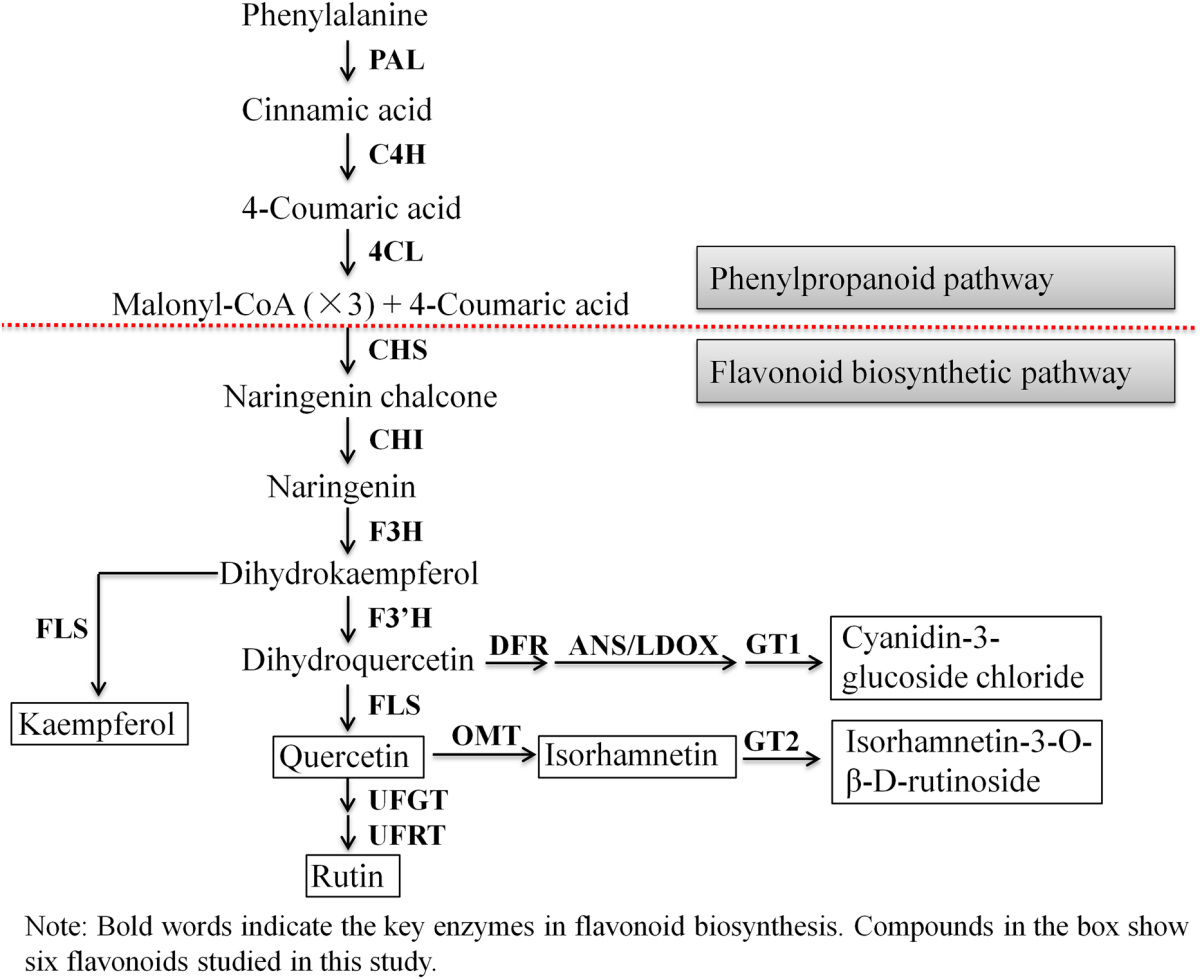
The biosynthesis pathway of 6 flavonoids in *A. roxburghii*.

**Figure 9:**
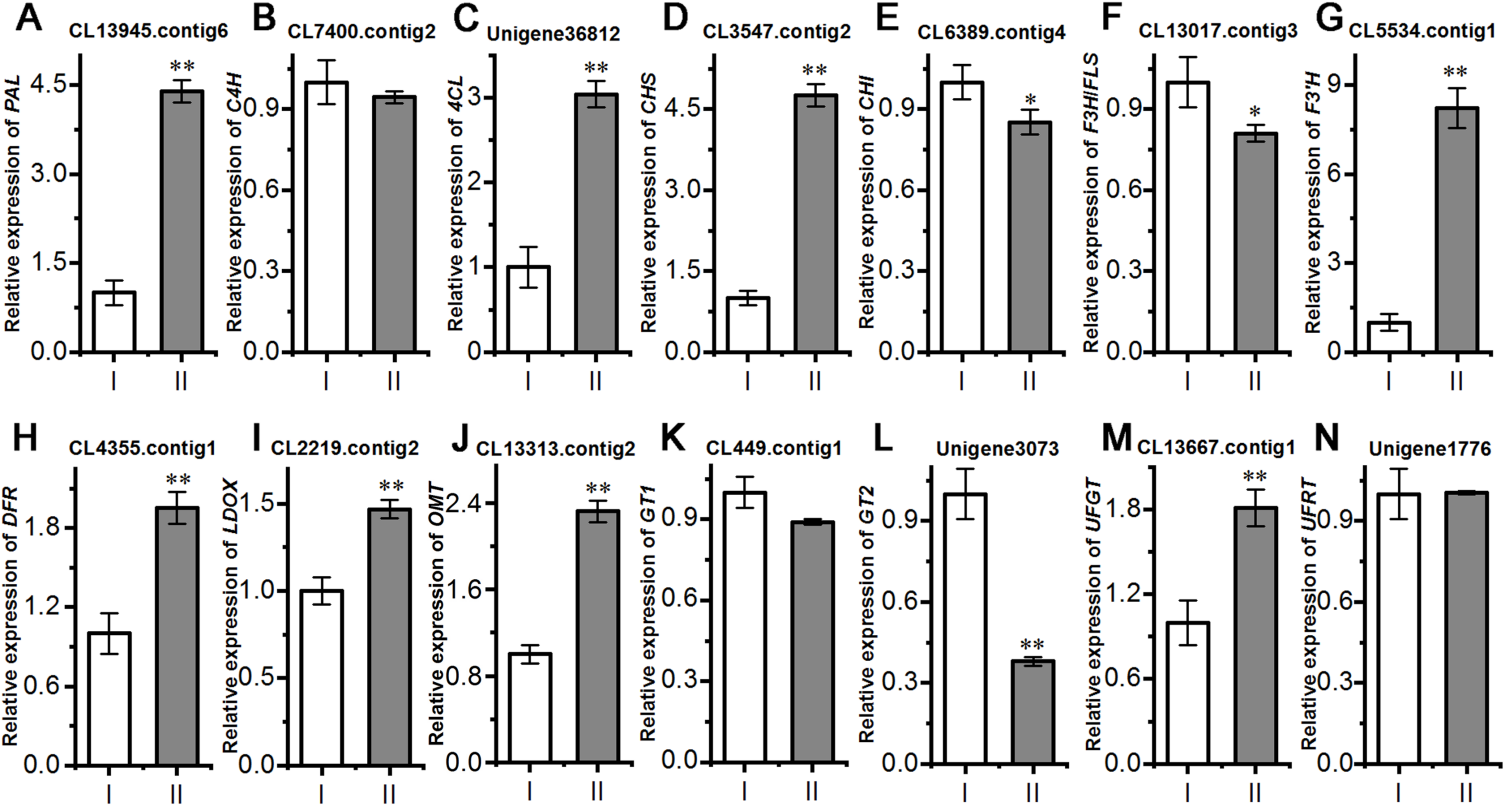
Comparision of the relative expression of 14 flavonoid biosynthesis genes in mycorrhizal *A. roxburghii* and non-mycorrhizal control. (A) Relative expression of *PAL*. (B) Relative expression of *C4H*. (C) Relative expression of *4CL*. (D) Relative expression of *CHS*. (E) Relative expression of *CHI*. (F) Relative expression of *F3H/FLS*. (G) Relative expression of *F3′H*. (H) Relative expression of *DFR*. (I) Relative expression of *LDOX*. (J) Relative expression of *OMT*. (K) Relative expression of *GT1*. (L) Relative expression of *GT2*. (M) Relative expression of *UFGT*. (N) Relative expression of *UFRT*. Each value is the mean of three replicates, and error bars indicate standard deviations. Statistical analysis of the data was performed by *t*-test. *P ≤ 0.05, **P ≤ 0.01.

Next, we evaluated the mechanisms of flavonoid accumulation in mycorrhizal *A. roxburghii*. The metabolic pathways of six common flavonoids in *A. roxburghii* are shown in Fig. 8, and 14 structural genes were found to be involved in its synthesis. We obtained nine phenylalanine ammonia lyase (*PAL*) genes, two cinnamate-4-hydroxylase (*C4H)* genes, twelve 4-coumarate CoA ligase (*4CL)* genes, seven chalcone synthase (*CHS)* genes, four chalcone isomerase (*CHI)* genes, fifteen flavanone 3-hydroxylase (*F3H)/flavonol synthase (FLS)* genes, ten flavonoid 3′-hydroxylase (*F3′H)* genes, seven dihydroflavonol 4-reductase (*DFR)* genes, seven leucoanthocyanidin dioxygenase (*LDOX)* genes, six O-methyltransferase (*OMT)* genes, twelve anthocyanin 3-O-glucosyltransferase (*GT1)* genes, seven 3-O-rutinoside-glucosyltransferase (*GT2)* genes, nine UDP-glucosyltransferase (*UFGT)* genes, and two 3-O-glucoside rhamnosyltransferase (*UFRT)* genes from transcriptome databases (Table S6). Among these genes, fourteen were analyzed using qRT-PCR to validate the accuracy of the transcriptome data. The chosen genes were all expressed in both sections to a greater or lesser extent (Fig. 9). *PAL* (CL13945. contig6), *4CL* (Unigene 36812), *CHS* (CL3547. contig2), *F3′H* (CL5534. contig1), *DFR* (CL4355. contig1), *LDOX* (CL2219. contig2), *OMT* (CL13313. contig2), and *UFGT* (CL13667. contig1) were significantly upregulated, while the genes *CHI* (CL6389. contig4), *F3H/FLS* (CL13017. contig3), and *GT2* (Unigene3073) were significantly downregulated in Section II compared with Section I. Furthermore, *C4H* (CL7400. contig2), *GT1* (CL449. contig1), and *UFRT* (Unigene1776) showed no significant changes between Section I and Section II. These validation results were consistent with the obtained transcriptome data, indicating that RNA-seq provided reliable data for the identification of key genes related to flavonoid metabolism in *A. roxburghii*.

### Contents of total and selected flavonoids of *A. roxburghii*

Using *A. roxburghii* with uninoculated AR2 as the control, the contents of total flavonoids and six known flavonoid monomers (quercetin, rutin, isorhamnetin, isorhamnetin-3-O-rutinoside, kaempferol, and cyanidin-3-glucoside chloride) in *A. roxburghii* were measured by colorimetric analysis and UHPLC-MS-MS, respectively. The recovery rates of quercetin (94.9%; relative standard deviation% (RSD%) = 2.7%), rutin (98.9%; RSD% = 11.2%), isorhamnetin (108%; RSD% = 4.3%), isorhamnetin-3-O-rutinoside (107.1%; RSD% = 4.4%), kaempferol (106.3%; RSD% = 2.6%), and cyanidin-3-glucoside chloride (105.0%; RSD% = 4.7%) were determined by comparing them with an external standard method. The multiple reaction monitoring parameters and calibrations for these standards are shown in Table 2. The *R*^2^ was higher than 0.9986. The lower limits of detection ranged from 1.53 nM to 24.41 nM, and the lower limits of quantitation were from 3.05 nM to 48.83 nM. As shown in Table 3, mycorrhizal fungi AR2 could significantly increase the accumulation of total flavonoids of *A. roxburghii*. Rutin, isorhamnetin, and cyanidin-3-glucoside chloride contents were significantly higher after inoculation with AR2 than in the control, showing increases of 62.59%, 260.00%, and 214.29%, respectively. In contrast, the yields of quercetin and isorhamnetin-3-O-rutinoside in Section II were lower than those in Section I. The content of kaempferol in Section I was 0.79 ± 0.01 nmol/g, although this compound was undetectable in Section II.

**Table 2:** MRM parameters and calibration of six flavonoids.

(A) MRM parameters of six flavonoids.						
Compound name	MRM parameters					
	Prec Ion	Prod Ion	Frag (V)	CE (V)	Polarity	Quantifier/Qualifier
Quercetin	300.9	178.8	160	20	Negative	Qulifier
Quercetin	300.9	150.8	160	20	Negative	Quantifier
Rutin	610.9	464.7	70	5	Positive	Qulifier
Rutin	610.9	303.0	70	15	Positive	Quantifier
Isorhamnetin	316.9	301.6	110	25	Positive	Qulifier
Isorhamnetin	316.9	152.8	110	35	Positive	Quantifier
Isorhamnetin-3-O-rutinoside	624.9	478.7	60	5	Positive	Qulifier
Isorhamnetin-3-O-rutinoside	624.9	316.9	60	15	Positive	Quantifier
Kaempferol	286.8	152.8	140	35	Positive	Quantifier
Kaempferol	286.8	120.8	140	35	Positive	Qulifier
Cyanidin-3-glucoside chloride	448.8	286.9	110	20	Positive	Quantifier/Qulifier

(B) Calibration of six flavonoids.				
Compound name	Calibration			
	Calibration curve	LLOD (nmol/L)	LLOQ (nmol/L)	*R*^2^
Quercetin	*y* = 4.1977*x* − 39.7382	12.21	24.41	0.9986
Quercetin				
Rutin	*y* = 16.4044*x* + 133.5839	3.05	6.10	0.9991
Rutin				
Isorhamnetin	*y* = 4.3920*x* − 5.6848	4.88	9.77	0.9987
Isorhamnetin				
Isorhamnetin-3-O-rutinoside	*y* = 17.5300*x* + 102.9274	6.10	12.21	0.9993
Isorhamnetin-3-O-rutinoside				
Kaempferol	*y* = 2.2613*x* − 23.0749	24.41	48.83	0.9992
Kaempferol				
Cyanidin-3-glucoside chloride	*y* = 57.0015*x* + 7.1925	1.53	3.05	0.9990

**Note:**

Prec Ion: precursor ion; Prod Ion: product ion; CE: collision energy.

**Table 3:** Flavonoid content of *A. roxburghii* inoculated with AR2 and uninoculated.

(A) Total flavonoid, quercetin, rutin, and isorhamnetin contents of *A. roxburghii* inoculated with AR2 and uninoculated.				
Sample	Total flavonoid (mg/g DW)	Quercetin (nmol/g DW)	Rutin (nmo/g DW)	Isorhamnetin (nmol/g DW)
I	11.25 ± 0.73^b^	2.74 ± 0.03^a^	154.89 ± 7.57^b^	0.25 ± 0.02^b^
II	15.42 ± 0.52^a^	1.88 ± 0.07^b^	251.84 ± 15.61^a^	0.90 ± 0.02^a^

(B) Isorhamnetin-3-O-rutinoside, kaempferol, and cyanidin-3-glucoside chloride contents of *A. roxburghii* inoculated with AR2 and uninoculated.			
Sample	Isorhamnetin-3-O-rutinoside (nmol/g DW)	Kaempferol (nmol/g DW)	Cyanidin-3-glucoside chloride (nmol/g DW)
I	978.20 ± 6.32^a^	0.79 ± 0.01^a^	0.21 ± 0.01^b^
II	805.48 ± 8.44^b^	0.00 ± 0.00^b^	0.66 ± 0.02^a^

**Note:**

Values represent mean ± SD (*n* = 3). Different letters represent significant differences (*P ≤* 0.05) on the same row according to the independent-sample *t* test. DW shows dry weight.

## Discussion

Early in the 1960s, Smith (1966, 1967) demonstrated that mycorrhizal fungi could directly provide nutrients to the host. Recently, research has indicated that mycorrhizal fungi could significantly promote the growth and development of orchid plants (Smith & Read, 2008; Zhao et al., 2014a). The symbiosis between orchids and mycorrhizal fungi has gradually attracted researchers’ interest. *Ceratobasidium* fungi are a major group of mycorrhizal fungi that form symbiotic associations with terrestrial orchids (Agustini et al., 2016; Rammitsu et al., 2019; Roberts, 1999). Wu, Huang & Chang (2011) reported that the leaf size and fresh weight in *Phalaenopsis* orchid plantlets inoculated with *Ceratobasidium* sp. AG-A (R02) showed significant increases. Cheng & Chang (2009) showed that the fresh weight and plant height of *A. formosanus* (line R) were obviously promoted by *Ceratobasidium* sp. AG-A symbiosis. In our study, AR2 was isolated from the roots of wild *A. roxburghii* and identified as a member of the genus *Ceratobasidium* by morphological observations and molecular techniques, and the cocultivation of *A. roxburghii* and AR2 was performed. Expectedly, the results indicated that the average fresh weight, dry weight, height, number of roots, and number of buds per plant were increased by 0.25 g, 0.18 g, 1 cm, 0.5, and 3, respectively, compared with those in the control. This suggests that AR2 shows great potential as a growth-promoting factor in the cultivation of *A. roxburghii*.

Active ingredients are the most important evaluation index of medicinal plant quality. Many studies have reported that mycorrhizal fungi, an essential biotic factor, affect the accumulation of flavonoids in medicinal plants. For example, *Acaulospora longula* was found to modulate the total flavonoid content in *Libidibia ferrea* (Dos Santos, Da Silva & Da Silva, 2017). Furthermore, flavonoid content increased significantly in *Amburana cearensis* seedlings treated with *Claroideoglomus etunicatum* (De Oliveira et al., 2015). Zhang et al. (2013) reported that *Mycena* sp. F-23 induced kinsenoside and flavonoid accumulation in *A. formosanus*. Consistent with these findings, the present results showed that AR2 could promote the accumulation of flavonoids in *A. roxburghii*, providing new insights into improving the quality of the product. In addition, fungal infection not only induces local changes in flavonoid contents in the roots, but also induces systemic changes in flavonoids in the same plant’s uninfected roots (Zhu & Yao, 2004). This mechanism may be related to the observation that AR2 induces flavonoid contents to maintain the symbiotic relationship in the roots, and that signal molecules are transported to uninfected sites to promote the production of flavonoids by regulating the expression of flavonoid biosynthesis-related genes. However, this is just speculation, and further studies are needed to verify this hypothesis.

With the development of molecular techniques and systems biology, the interaction between fungi and orchids has been studied by transcriptome and metabolome analyses. Zhao et al. (2014b) characterized the root transcriptome of *Cymbidium hybridum* inoculated with different mycorrhizal fungi and found that cell wall modification, reactive oxygen species detoxification, defense-related phytohormone and phosphate transport were co-induced in all symbiotic interactions. Li et al. (2017a) proved that the mycorrhizal fungus MF23 stimulated dendrobine accumulation in the stems of *D. nobile* by regulating the expression of genes involved in the mevalonate pathway through a combination of RNA-seq, qRT-PCR, and gas chromatography. Transcriptome analyses in our study revealed 9,645 DEGs between the control and treatment groups and a large proportion of the DEG KEGG pathways were involved in metabolic pathways and biosynthesis of secondary metabolites. LC-MS/MS and qRT-PCR indicated that AR2 induced the accumulation of flavonoids by regulating the expression of related genes in the flavonoid synthesis pathway. These results provide a basis for further studies of the molecular mechanisms through which AR2 promotes flavonoid production in *A. roxburghii*.

Anthocyanins, including cyanidin, pelargonidin, delphindin, peonidin, petunidin, and malvidin, are water-soluble natural pigments that directly affect color formation in different parts of plant tissues (Cabrita, Fossen & Andersen, 2000). Their accumulations in plants have been suggested to be associated with the presence of mycorrhizal fungi, which could regulate the activity of related enzymes or the expression of related enzyme genes in the anthocyanin synthesis pathway. For example, Harrison & Dixon (1994) reported that *Glomus versiforme* colonization caused the induction of PAL activity in inoculated *Medicago truncatula* plants. Mollavali et al. (2016) also reported that mycorrhizal inoculation increased PAL activity in onions. Moreover, PAL activity was positively correlated with anthocyanin content (Tan et al., 2014). In addition, *F3′H*, known as the “red gene”, directly affects the type of anthocyanins (Wang et al., 2014). DFR catalyzes the conversion of dihydroquercetin into colorless leucoanthocyanins and anthocyanin synthase (ANS) converts colorless anthocyanins into colored anthocyanins (Li et al., 2012). In this study, we found that leaf color differed between the control (green) and treatment group (green plus red). Wang et al. (2014) reported that the color was related to the presence of cyanidin-type (pink to red) anthocyanins. Based on this, cyanidin-3-glucoside chloride was used as the detection index. As expected, the contents of cyanidin-3-glucoside chloride were approximately 3-fold higher in mycorrhizal *A. roxburghii* than nonmycorrhizal organisms. Furthermore, the results indicated that AR2 triggered the expression of *PAL*, *F3′H*, *DFR*, and *ANS* (resulting in 4.39-, 8.21-, 1.95-, and 1.47-fold increases, respectively). Based on the above results, the content of flavonoids, including anthocyanin in *A. roxburghii*, was obviously accumulated after inoculation with AR2. Further experiments are needed to elucidate the mechanisms involved in these effects.

## Conclusions

The mechanisms mediating changes in flavonoid levels in the medium from *A. roxburghii* cocultured with the AR2 isolate were analyzed in this study. The isolated fungus AR2 not only promoted the growth of *A. roxburghii* in vitro but also enhanced flavonoid content by inducing the expression of related structural genes. The current findings will improve our understanding of the mechanisms that mycorrhizal fungi promote flavonoid accumulation in *A. roxburghii* and provide basic data for further research.

## Supplemental Information

10.7717/peerj.8346/supp-1 Supplemental Information 1 Primer designed for qRT-PCR.

10.7717/peerj.8346/supp-2 Supplemental Information 2 R, G, B value of leaf color from the non-mycorrhizal and mycorrhizal *A. roxburghii*.

10.7717/peerj.8346/supp-3 Supplemental Information 3 Summary of sequences analysis.

10.7717/peerj.8346/supp-4 Supplemental Information 4 Assembly results.

10.7717/peerj.8346/supp-5 Supplemental Information 5 BLAST analysis of non-redundant unigenes against public databases.

10.7717/peerj.8346/supp-6 Supplemental Information 6 The major structural genes involved in flavonoid biosynthesis from transcriptome database.

10.7717/peerj.8346/supp-7 Supplemental Information 7 DEGs in KEGG pathway.

10.7717/peerj.8346/supp-8 Supplemental Information 8 The raw data of effects of mycorrhizal fungi AR2 on growth of *A. roxburghii*..

10.7717/peerj.8346/supp-9 Supplemental Information 9 The raw data of total flavonoid content and 6 flavonoid content.

## Additional Information and Declarations

### Data Availability

The following information was supplied regarding data availability: Data is available at GenBank: MN068847 and PRJNA562071.
